# N-Screen Aware Multicriteria Hybrid Recommender System Using Weight Based Subspace Clustering

**DOI:** 10.1155/2014/679849

**Published:** 2014-07-24

**Authors:** Farman Ullah, Ghulam Sarwar, Sungchang Lee

**Affiliations:** Department of Information & Communication, Korea Aerospace University, Goyang 412-791, Republic of Korea

## Abstract

This paper presents a recommender system for N-screen services in which users have multiple devices with different capabilities. In N-screen services, a user can use various devices in different locations and time and can change a device while the service is running. N-screen aware recommendation seeks to improve the user experience with recommended content by considering the user N-screen device attributes such as screen resolution, media codec, remaining battery time, and access network and the user temporal usage pattern information that are not considered in existing recommender systems. For N-screen aware recommendation support, this work introduces a user device profile collaboration agent, manager, and N-screen control server to acquire and manage the user N-screen devices profile. Furthermore, a multicriteria hybrid framework is suggested that incorporates the N-screen devices information with user preferences and demographics. In addition, we propose an individual feature and subspace weight based clustering (IFSWC) to assign different weights to each subspace and each feature within a subspace in the hybrid framework. The proposed system improves the accuracy, precision, scalability, sparsity, and cold start issues. The simulation results demonstrate the effectiveness and prove the aforementioned statements.

## 1. Introduction

N-screen (multidevices) spurred the research in this field of study in order to provide a mechanism to share and move the content among devices having different screen sizes, resolutions, and network access interfaces [1, 2]. The N-screen phenomenon started a few years ago when the computer became a household staple and picked up speed with the laptop and will become very colloquial when smart devices such as smart phones, iPads, and tablets achieve a critical mass among affluent consumers. According to the multiscreen survey report by IAB [3], 77% of people in the USA are using three screens while 69% of people in the USA are using four screens. The number of content and smart devices is increasing on the user side, so it is an issue for a user to find the contents that his device and access network are capable of displaying and streaming. 65% of smartphone users say that most of the sites do not work well for their devices [3]. We propose a new recommender system that accounts for user device and access network capabilities as well as content preferences and demographics.

The explosion of communication technologies and multimedia-capable devices is influencing the ways in which users expect to interact with the content. The heterogeneous nature of device attributes and access network conditions present new questions in the realm of user experience with the content and quality of service [4]. Catellier et al. in [4] analyzed the impact of mobile devices and usage location on perceived multimedia quality, and according to them users have different experience and satisfaction levels when they shifted from one device or access network to another. A psychophysical experiment was carried out in [5] to measure the impact of user opinion about the perceived image quality on mobile-phone displays by changing different image parameters, and according to them excellent image display affects user opinion about content. To improve user experience with multimedia content, we propose N-screen aware recommendations by introducing the user device profile collaboration agent (UDPCA), user device profile collaboration manager (UDPCM), and N-screen control server (NCS).

Recommender systems (RSs) are invaluable tools to help users in handling information overload, as the number of items (e.g., products, news, movies, and music) is growing exponentially. RSs are software applications providing suggestions for items to be of use to a user using either his history of interests, contextual information, social interactions, or his behavior similarities to other users. The suggestions are based on different decision making algorithms, which turn the user preferences into predictions of possible future user likes and interests. The computational approach for a recommendation is to select the objects favored by other users that are similar to the target user's profile. Typically, RSs compare some characteristics of users' profiles or items to find similar users or items and predict the rating that the active user would assign to an item. The existing RSs find similar users and use their watched content as a candidate for recommendations. The existing systems had an issue that one user may like content at night but due to the similarity with another user the system can recommend it in the daytime. We consider temporal information with user ratings to find which user likes which content at which time.

Due to exponential growth and development in data generating sources, RSs are entering into every field of life to help users get useful information from an overabundance of data. Social networks like http://www.facebook.com analyze user profiles and contacts to connect them with new friends. The online product sellers like http://www.amazon.com carefully watch purchased transactions to suggest other products. RSs not only help to recommend products but also increase cross-selling by suggesting related or complimentary products to a customer [6]. Most of the existing RSs get a single value as a preference for an item from a user. The single-rating RSs are successful in many application areas; however, multicriteria recommender systems are also required. The majority of organizational decisions are multicriteria problems. For example, power transmission routes in cities consider cost, health, reliability, and the importance of the area. The existing multicriteria RSs use *K*-means for clustering similar users and assign the same weight to each feature. We present a unified framework, which incorporates the user N-screen information with temporal aware multirating and user demographics, and consider each of the three as a subspace. Instead of the *K*-means algorithm, we propose an individual feature and subspace weight based clustering algorithm, which assigns different weights to each subspace and individual feature within a subspace.

We organize the paper as follows.  Section 2 explains related work regarding recommender systems.  Section 3 describes the architecture and overall framework, and Section 4 describes details of each component in the framework.  Section 5 is about the simulation and results. Finally we conclude our work in Section 6.

## 2. Background and Related Work

The research closely related to our work is conventional recommendation and personalization systems of online movies or videos on demand. The central parts of the RSs are the recommendation algorithms, and based on recommendation algorithms they can be classified as collaborative filtering [7–12], content-based [11–13] graph theory, network structure based [10, 14], rule-based approach [15, 16], and hybrid approach [15, 17] based recommender systems.

Collaborative filtering (CF) depends on user opinions such as rating information. CF methods store enough information and use this for finding similarities with the target user and recommend an item based on the similarity. Most of the CF algorithms consider that a sufficient collection of users or items profiles is available and it recommends items, matching to user profiles. In other words, the CF considers the user interests and profile instead of item's attributes and content. The CF algorithms are of three types: memory based [10, 12, 18–20], model-based [10, 19, 20], and the hybrid approach [15, 17, 19]. In memory-based CF, algorithms use the entire database for each recommendation. Breese et al. [21] used some partial information about the active user and the weighted average of opinions of other users from history to predict votes for the active user. The *K*-nearest neighbor (*KNN*) algorithm best describes this mechanism. The* KNN* algorithm finds the *K*-similar neighbors and uses their ratings to find the prediction value for the object to the active user [20]. The memory-based CF increases the accuracy at the cost of processing and scalability. The model-based algorithms construct the preferences model from the user opinions, which extract some information from the user opinion dataset and make a model for recommendations instead of using the whole dataset.

Content-based filtering (CBF) recommends items to the active users based on the content similarity with what users watched or experienced before. Unlike CF, the CBF algorithm built a profile by analyzing the contents of the items that active user had rated in the past. The content-based recommender systems discover semantically related contents and keywords. The main problem with these algorithms is that they need structured information and they are not useful for unstructured data such as videos, audios, and images.

Most of the RSs get a single value for user preference about an item, which represents the preference of user *u* on item *i*. The recommender system must be able to understand not only what people like but also why and when they like it. In decision theory, the field of multicriteria decision analysis (MCDA) emerged from the fact that most of the real world problems are naturally multidimensional [22]. Based on MCDA, numerous multirating recommender systems are proposed. Lakiotaki et al. [23] proposed *K*-means based multicriteria user modeling in recommender systems. They model the user rating and use *K*-means clustering to group the similar users for similarity and recommendation. A multicriteria RS is proposed in [16] for interval scaled rating in order to find the weights to convert the multirating Yahoo! movies data to a single-rating. Nilashi et al. [24] proposed hybrid recommendation approaches for multicriteria CF using adaptive neurofuzzy inference systems and self-organizing map. The issue with most of the proposed multicriteria recommendation techniques is that they consider the *K*-means which assigns equal weight for all features. A two-level variable weighting clustering algorithm is proposed in [25], and Guo et al. [26] proposed a soft subspace and improved feature weight self-adjustment mechanism for higher dimension data. Considering that a user has multirating preferences, rated multiple programs on various devices, and has demographics, we suggest an IFSWC to assign different weights to subspace and feature. The proposed clustering algorithm objective function considers the separation among clusters' centers to converge in a small number of iterations.

CF has problems of cold start issues and sparsity, while CBF needs structured information as well as having cold start issues. In order to solve these issues, a hybrid approach is used. The hybrid RSs combine CF with the CBF or any other information such as demographics. Hybrid RSs merge two or more recommendation techniques to improve the accuracy and combine the predictions obtained from different methods either by a simple linear combination [27] or by a complex method such as Markov chain Monte Carlo [28]. The hybrid RSs combine the techniques [10] by four methods: (1) finding predictions separately and then combining them for single prediction, (2) integrating some characteristics of CBF into a CF process, (3) integrating some characteristics of CF into CBF, and (4) constructing a unified model that integrates the characteristics of both CF and CBF. The hybrid RSs improve the sparsity, cold start issue, diversity, and scalability of the CF and CBF. CF has complexity because finding similar users and recommending an item are complex when the number of users and items is large. To overcome the complexity and cut system overload Xue et al. [29] suggested a two-step process which clusters similar users offline and then uses the Pearson correlation coefficient for item rating prediction.

We can summarize the contribution of our research in the field of recommendation as follows. (1) It can be applied in any field of recommendation, but it is highly applicable in the field of online movies or programs recommendation because it recommends the contents by considering the user device's static and dynamic profile. (2) It uses temporal information to find out which user likes which content at which time, for example, if a user likes short length videos during his office time and drama and family movies with his family at night time. (3) We use a hybrid unified model that incorporates the user rating information, demographics, and users' N-screen information. (4) The proposed individual feature and subspace weight based clustering produces good quality of clusters as compared to simple *K*-means used in many RSs. (5) We improve the sparsity and cold start issues using a hybrid framework and precision/recall by considering temporal information.

## 3. The Proposed N-Screen Aware Multicriteria Hybrid Recommender System

**Figure 1:**
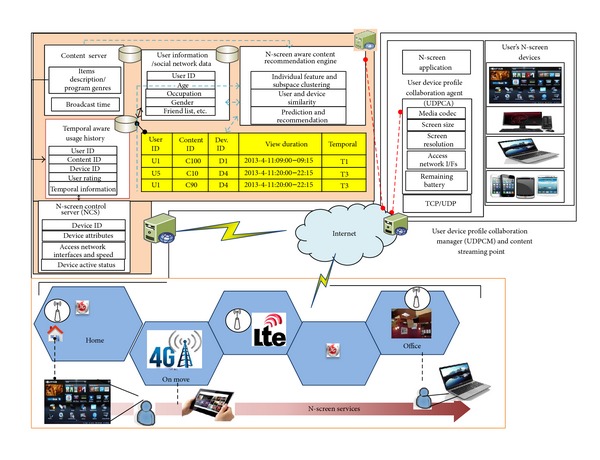
Architecture of N-screen aware multicriteria hybrid recommender system.

The heterogeneity of devices and access network conditions necessitate an N-screen content aware recommendation system in order to ensure that the user N-screen device and access network are capable of streaming and displaying with the user's preferences. This section presents the architecture of the proposed N-screen aware multicriteria hybrid recommender system.  Figure 1 shows the overall architecture of the proposed system. For N-screen aware recommendations support, we introduce the N-screen application, user device profile collaboration agent (UDPCA), user device profile collaboration manager (UDPCM), and N-screen control server (NCS). Since in N-screen services the user can access the content through heterogeneous devices having various screen sizes, the N-screen application provides a web-based dynamic user interface that adapts the content according to the N-screen layout. The N-screen application provides a login, the recommendation list, the streaming content display, and interface to provide user opinion about the streaming content. The details of the functional and logical nodes of the UDPCA, UDPCM, and NCS are as follows.

**Figure 2:**
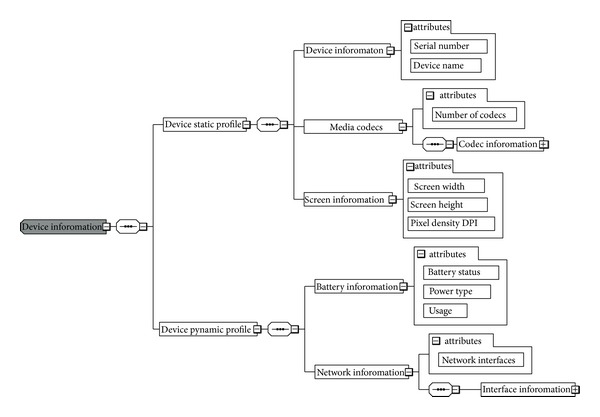
Schema for user N-screen device profile attributes.

*UDPCA*. It is an application plugin that runs on the user N-screen device to acquire the static and dynamic profile of the device.  Figure 2 shows the XML schema for the user device profile. The device static profile includes the user device information like storage, screen information, and media codec. The user device dynamic profile includes the resource type, that is, memory and CPU usage, remaining battery time, and the current connected network interface and its bandwidth. The UDPCA provides the device attributes on the request of the UDPCM using the RESTful URIs.

*UDPCM*. The user device profile collaboration manager uses the RESTful web-based architecture to manage the acquisition of the device's static and dynamic profile of the user's N-screen. The static profile is acquired once when the user registers his device. The dynamic profile is acquired during the content streaming and uses the average and normalized value for the available network bandwidth and remaining battery time. UDPCM runs either at the smart TV setup box or at the streaming distribution point. We are using temporal information and divided the time range of the day in four ranges. The UDPCM updates the N-screen control server information at the end of each temporal range.

**Figure 3:**
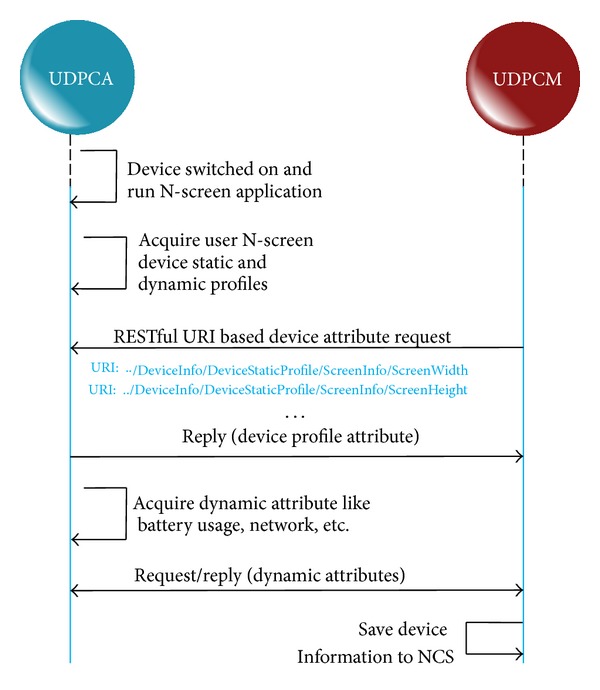
Protocol for the acquisition of user's N-screen device profile.

*NCS*. The NCS maintains the repository of user N-screen devices and the status of each device, whether it is active or not. NCS stores both the static and the dynamic profile of the user N-screen devices.  Figure 3 shows the protocol and procedure for the user's N-screen device profile acquisition. If the user is a new user, after registration he is directed to register his N-screen devices. If the user is an existing user, so after launching the N-screen application, the UDPCA acquires the device profile and the UDPCM request for device attributes using the Restful URIs. Although a number of N-screen attributes can be used, we consider the screen information, remaining battery time, and access network in this work.

In conventional RSs, the systems use either the users similarity or items similarity, and based on this they can recommend the content that a user watched at night to another user at the daytime. However, the user's preferences and streaming content display devices change with the time of the day; for example, at night the user prefers to watch drama with his family at large screen but at the office he prefers to watch short video clips on his smart phone. To improve the accuracy of N-screen aware recommendation and user experience with content, we introduce the temporal aware usage history and divided the time of the day in four temporal ranges. In the recommendation architecture with N-screen information and temporal awareness, we introduce individual feature and subspace weight based clustering (IFSWC) to assign a different weight to each subspace, that is, user preferences, N-screen information and user demographics, and a weight to each feature within a subspace.

## 4. The Proposed N-Screen Aware Multicriteria Hybrid Recommender System Using IFSWC Scheme

**Figure 4:**
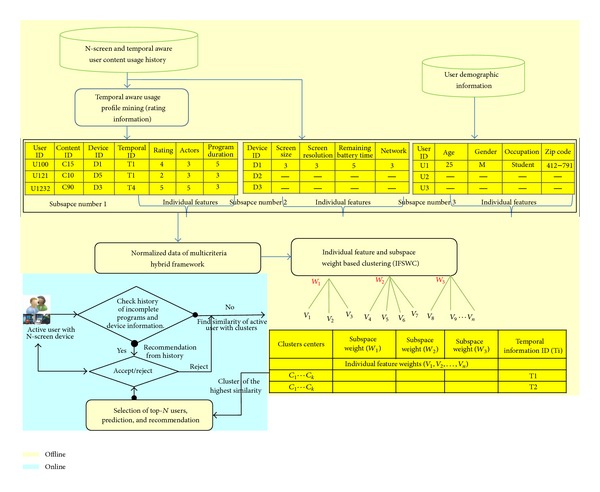
Algorithm/flow chart of N-screen aware multicriteria hybrid recommender system using weight based subspace clustering.

This section presents our proposed scheme of N-screen aware recommendation.  Figure 4 shows the flow chart of the proposed recommendation scheme. We divided our algorithm in two phases, that is, online and offline phase. At the end of each temporal range, we mine the user history in that temporal period, extract the user preferences, and update the clusters centroid offline. In the online phase, we measure the similarity of the target user with the clusters centroid and select the cluster having the highest similarity. Considering the top-*N* most similar users from the cluster, we predict the item features and recommend the top-*N* items considering the user active device profile. We focus in this section on the components of the proposed scheme but mainly on the IFSWC and recommendation algorithms.

### 4.1. Temporal Aware User Profile and Multicriteria Hybrid Framework

We characterized the user profile by his preferences on an item having multirating, demographics, and N-screen device profile. The user provides his demographics information and number of N-screen devices during service registration. We acquire the user device profile as we described in Section 3. It is better if the user provides his preferences about the content explicitly, but, due to different interaction mechanisms with user N-screen and the small screen display of mobile devices, it is hard to get an explicit rating from a user. We implicitly find the user preferences about content from the user temporal aware usage history, considering only those contents that the user watched 25% of the total time during transmission in a specific temporal range for a certain length of history. The preference *p* of a user *u* for an item/program *m*, device *d*, and temporal information *t* can be found by using the following equation:

(1) $$\begin{array}{c} p\left(u,m,d,t\right)=\frac{\sum_{b=1}^{N_{b}}T\left(b,m,d,t\right)}{\sum_{b=1}^{N_{b}}T\left(b,m\right)}, \end{array}$$

where *N*_*b*_ is the number of times a program *m* is broadcasted, *T*(*b*, *m*, *d*, *t*) is the user program watched duration during a broadcast, and *T*(*b*, *m*) is the total time of a broadcast. If a user has not provided an explicit rating about a program, by using (1) we find it implicitly.

A unified hybrid framework is used that incorporates the user temporal aware preferences with his demographics and N-screen information. The framework provides the following advantages. (1) Using temporal information, it considers only effective users at a specific time range. (2) The user rating has a smaller size considering only effective items and temporal information to minimize the computational complexity, so it requires less time to find similar users compared to the traditional collaborative filtering. (3) Since the model incorporates and fuses three different sources of data, we propose IFSWC to assign different weights to each feature and subspace to improve the clustering accuracy.

### 4.2. Individual Feature and Subspace Weight Based Clustering (IFSWC)

This paper suggests an IFSWC to assign different weights to each subspace and individual feature to improve the clustering quality. For effectiveness and computational efficiency, we cluster similar users considering temporal information, that is, specific time range. Since our algorithm fuses different sources of data, we embed the weight mechanism in the *K*-means clustering algorithm to assign different weight. The objective function for the proposed IFSWC clustering algorithm is

(2) F(U,C,W,V)=(∑u=1U∑ k=1Kλu,k∑g=1G∑ n=1Nwgvn((∑m=1M(xk,m,g,n−ck,m,g,n)2)           + (x^u,g,n−c^k,g,n)2))1/2 −1K(∑g=1G∑ n=1N∑ k=1k≠k′Kwgvn((∑m=1M(ck,m,g,n−ck′,m,g,n)2)           + (c^k,g,n−c^k′,g,n)2))1/2

subject to

(3) ∑k=1Kλu,k=1; where  λu,k∈{0,1};foru=1,2,…,U; k=1,2,…,K,g=1,2,…,G;m=1,2,…,M; n=1,2,…,N,∑n=1Nvn=1; where  vn∈[0,1],∑g=1Gwg=1; where  wg∈[0,1].

The objective function *F*(*U*, *C*, *W*, *V*) shows the distance of the user from the cluster centroid and assigns it to the cluster having less distance from the cluster center. Nomenclature Section shows the variables description we used in this work. The cluster membership of a user is decided by using the membership decision equation

(4) $$\begin{array}{c} \lambda_{u,k}=\{\begin{array}{cc} 1; & \text{if}\;F\left(c_{k,g,n},w_{g},v_{n}\right)<F\left(c_{l,g,n},w_{g},v_{n}\right)\\ & \text{for}\;l=1,2,\ldots K,\;\;l\neq k\\ 0; & \text{otherwise}. \end{array} \end{array}$$

We update the feature center of user multirating and N-screen attribute in a cluster by using (5). Since some of the programs are not corated among cluster users, so we add them in the cluster while for corated programs we find the average value using (5). The demographic feature center is updated by (6)

(5) ck,g,n=∑u=1U∑m=1Mλu,kxu,m,g,nM∑u=1Uλu,k,

(6) c^k,g,n=∑u=1Uλu,kx^u,g,n∑u=1Uλu,k.

The weight shows the subgroup and feature importance and helps to achieve the proposed clustering objective by minimizing the distance of separation within the cluster objects and maximizing the separation among the clusters' centers. In (2), we also subtract the separation among the clusters in order to converge the cluster objective in smaller number of iterations. By increasing the feature and subgroup weights of important features, we can achieve the cluster objective easily.

Let *a*_*n*_ and *b*_*n*_ represent separation distance within cluster objects and among the clusters. Equations (7) and (8) show the separations, respectively:

(7) an=(∑u=1U∑ k=1Kλu,k∑g=1Gwgvn((∑m=1M(xk,m,g,n−ck,m,g,n)2)          + (x^u,g,n−c^k,g,n)2))1/2 −1K(∑g=1G∑ k=1k≠k′Kwgvn((∑m=1M(ck,m,g,n−ck′,m,g,n)2)          + (c^k,g,n−c^k′,g,n)2))1/2,

(8) bn=(∑k=1K||ck||∑g=1Gwgvn((∑m=1M(ck,m,g,n−zg,n)2)         + (c^k,g,n−zg,n)2))1/2,

where *z*_*g*,*n*_ is the global mean of a feature *n* in a subgroup *g*. We use the self-adjustment mechanism of feature and group weight proposed by Tsai and Chiu in [30]:

(9) $$\begin{array}{c} v_{n}^{\left(s+1\right)}=\frac{1}{2}\left(v_{n}^{s}+\Delta v_{n}^{s}\right),\\ w_{g}^{\left(s+1\right)}=\frac{1}{2}\left(w_{g}^{s}+\Delta w_{g}^{s}\right), \end{array}$$

where

(10) $$\begin{array}{c} \Delta v_{n}^{s}=\frac{b_{g,n}^{s}/a_{g,n}^{s}}{\sum_{g=1}^{G}\sum_{n=1}^{N}\left(b_{g,n}^{s}/a_{g,n}^{s}\right)}; \end{array}$$

(11) $$\begin{array}{c} \Delta w_{g}^{s}=\frac{\sum_{n=1}^{N}\left(b_{g,n}^{s}/a_{g,n}^{s}\right)}{\sum_{g=1}^{G}\sum_{n=1}^{N}\left(b_{g,n}^{s}/a_{g,n}^{s}\right)}. \end{array}$$

Using the above equations, we find the weights for each feature and subgroup and feed this information back to the clustering algorithm for the next iteration.  Algorithm 1 shows the procedure for the proposed IFSWC based clustering algorithm.

### 4.3. Content Recommendation to User's N-Screen Device

The N-screen aware recommendation ensures that the user's N-screen device and access network are capable of displaying and streaming. In the proposed architecture, the NCS maintains the user's N-screen device attribute repository and the active status of the device. The system acquires the user's N-screen device dynamic profile and ensures that the user's N-screen is already registered and its static profile is in the NCS. Based on the active status of the user's N-screen, we find the dynamic profile of each of these devices. To recommend the content to the user's N-screen, we retrieve the active user experienced programs with the N-screen information of the active devices from the user data. In this data, we replace the dynamic profile attributes with the current dynamic attributes of the device and access network. Although we can provide a recommendation to the user on multiple active N-screen devices, the scope of this work is to consider a single active N-screen device. The following steps are involved in the recommendation phase: (1) selecting the cluster of the highest similarity with the active user, finding users from that cluster having higher similarity with the active user, and using their programs as a candidate for recommendation, (2) finding a prediction about an item to select the candidate items for recommendations, and (3) selecting the top-*N* content as a recommendation to the user ensuring that the device and access network are capable of displaying with user preferences.

#### 4.3.1. Selecting Cluster and the Highest Similarity Users

As in Section 4.1, we explained that users are clustered in a specific temporal range. Considering the active user temporal information, we use the clusters of that temporal range to find a cluster having the highest similarity with the active user. Furthermore, we select the top-*N* most similar users from that cluster in terms of user rating, N-screen information, and demographics. The proposed system improves the computational delay by considering the temporal information. The algorithm to find the similar preference users is shown in Algorithm 2.

#### 4.3.2. Prediction and Selection of Candidate Items/Programs

We consider items of the top-*N* users that the target user has not watched previously as candidate items for recommendations. Equation (12) predicts the item and N-screen feature for the active user:

(12) $$\begin{array}{c} P\left(u,\alpha_{j}\right)=\bar{R_{u,j}}+\frac{\sum_{i\in U}\text{usersim}\left(u,i\right)\left(R_{i(\alpha ,j)}-\bar{R_{i(j)}}\right)}{\sum_{i\in U}\text{usersim}\left(u,i\right)}, \end{array}$$

where *P*(*u*, *α*_*j*_) is prediction on feature *j* of item and device attribute for target user *u*, *U* denotes the most similar preference users to the target user, and *α* is a feature vector that shows the program and the N-screen on which the user watched the program. *R*_*i*(*α*,*j*)_ are the rating of user *i* for feature *j* of item/device *α*, $\bar{R_{u,j}}$ is average rating of target user for *j*, and $\bar{R_{i(j)}}$ is average rating of user *i* for feature *j*.

#### 4.3.3. Top-*N* Content Recommendations to User's N-Screen Device

After selection and finding predictions on candidate items for recommendation, we have to select the top-*N* contents to recommend them to the user. If the user has a history, that is, previously watched content, then we find the average rating of each feature and let *T*_*u*_ be the set of programs that target user *u* has experienced on the active N-screen device. The average rating given to a feature *j* by the target user *u* is given by $\bar{r_{j}}=\left(1/\left|T_{u}\right|\right)R_{\alpha ,j}$. Equation (13) finds the distance of the program multirating and the used N-screen attributes to watch this program of the target user with the candidate items experienced on that device for the recommendation:

(13) $$\begin{array}{c} d_{\bar{r}r_{c}}=\sqrt{\overset{N_{r}}{\underset{j=1}{\sum }}{\left(r_{c,j}-\bar{r_{j}}\right)}^{2}}, \end{array}$$

where $d_{\bar{r}r_{c}}$ is the distance and, having low value of $d_{\bar{r}r_{c}}$, we recommend that program. *N*_*r*_ shows the number of features (program multirating and the N-screen attributes on which that program is watched). If the user is a new user and has no experienced items in his history, then $\bar{r_{j}}=0$, so we recommend the content from the user that has the highest demographics and device similarities.

## 5. Evaluation and Results

To evaluate the effectiveness of the proposed recommender system, we use the multicriteria movies data collected from Yahoo Movies! [31] and modify it by inserting the temporal information and the N-screen attributes [32]. The modified dataset includes the user preferences about the program story, actors, and length of the program; the N-screen information of screen size, remaining battery time, and available access network speed; user demographics of gender and age; and temporal information. We cluster the users in 15 clusters. The weights we achieved for the subspaces are {0.3792,0.3770,0.2437} and for individual feature are {0.1381, 0.1201, 0.1210, 0.1200, 0.1302, 0.1268, 0.1097, and 0.1340}. The performance of the proposed recommender system is measured in terms of mean absolute error (MAE) and precision/recall. We consider the users that had rated at least five movies in common with the same N-screen device and divided the dataset into 80% of training and 20% as test data and use 10-fold cross-validation of results.

### 5.1. Mean Absolute Error (MAE)

**Figure 5:**
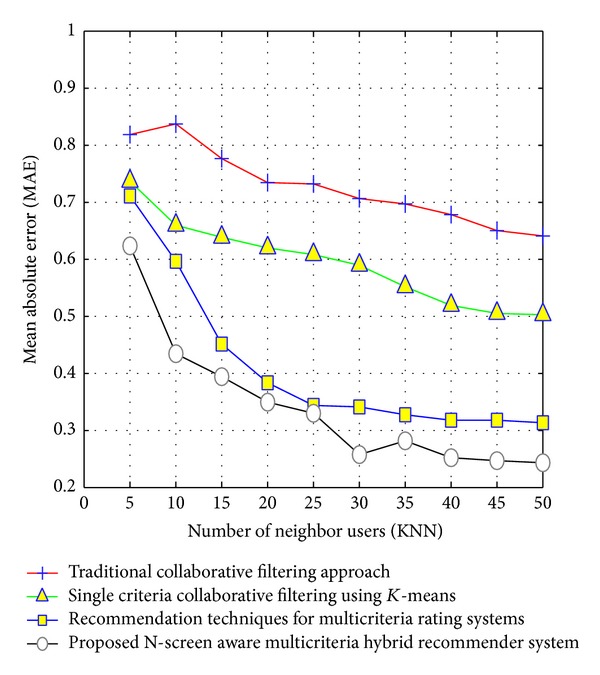
Comparison of MAE performance measure.

The most commonly used metric for finding the accuracy of a recommender system is the MAE. The MAE finds the deviation of predictions on items generated by recommender system from the true rating values, and having low MAE value means better prediction and recommendation accuracy. If *n* is the top-*N* content recommended to the user *u*, then MAE is given by the following equation:

(14) $$\begin{array}{c} \text{MAE}=\frac{1}{\left(n*J\right)}\overset{n}{\underset{i=1}{\sum }}\overset{J}{\underset{\;j=1}{\sum }}\left|r_{i,j}-r_{i,j}^{\prime }\right|, \end{array}$$

where *r*_*i*,*j*_ is the actual value that the user assigned to a feature *j* of an item *i* and *r*_*i*,*j*_′ is its predicted value. We recommend the top-10 items/programs to the target user. We compare the proposed system with the new recommendation techniques for the multicriteria rating system proposed (MCRS) in [33], traditional collaborative filtering (TCF) of [7], and single criteria collaborative filtering using *K*-means clustering (SCCFK-means) of [34].  Figure 5 shows the MAE performance measure of the proposed scheme with other schemes of recommendation. The MAE measures in this paper only consider the user preferences about an item and do not consider the N-screen and user demographics attributes to get an effective comparison. In (14), *J* = 3 for the proposed system, *J* = 4 for MCRS, and *J* = 1 for TCF and SCCFK-means. The proposed system has better performance and lower value of MAE for the following reasons. (1) The proposed IFSWC groups the most similar users in a cluster having minimum separation within a cluster and higher among clusters and improves the accuracy to find similar users. (2) We use multicriteria, that is, user multirating, N-screen attributes, and demographics to find similar users. (3) We assign higher weight based on the importance of a subspace and an individual feature within a subspace that improves the clustering accuracy and in the long run the prediction accuracy.

### 5.2. Precision and Recall

In information retrieval, precision and recall are the most popular metrics for evaluation of the system. Precision means the exactness and recall means the completeness [35]; that is, precision shows the relevant items in the top-*N* and recall shows that the retrieved top-*N* are relevant. Precision and recall are given, respectively, by

(15) $$\begin{array}{c} \text{precision}=\frac{\left|\text{test}⋂\text{Top}\_N\right|}{\left|\text{Top}\_N\right|}, \end{array}$$

(16) $$\begin{array}{c} \text{recall}=\frac{\left|\text{test}⋂\text{Top}\_N\right|}{\left|\text{test}\right|}. \end{array}$$

**Figure 6:**
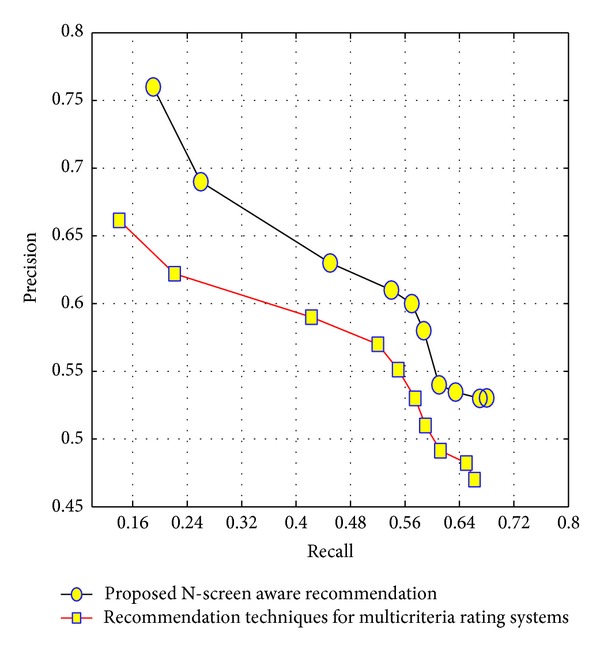
Performance comparison of precision/recall.

We compute the precision and recall by considering the top-20 nearest neighbor users to the target user by recommending the items (*recommendation*_*set* = {5,10,15,20,25,30,35,40,45,50}).  Figure 6 shows the performance comparison of the precision and recall of the proposed system with the MCRS. The proposed system performs well in terms of precision and recall because (1) we introduce temporal information in the proposed system that helps to predict which content the user prefers at which time, which improves the precision/recall. (2) The IFSWC improves the cluster quality and produces the most similar nearest neighbor users.

## 6. Conclusion

This paper proposes a new recommender system for user N-screen aware recommendations that incorporates the user N-screen device attributes like screen size, access network speed, and remaining battery time and temporal aware usage information that are not considered in previous recommender systems. The proposed system guarantees that a user can watch the content on different devices, at different times, and in different locations with his preferences. The proposed recommender system introduces the UDPCA, UDPCM, and NCS to acquire the user N-screen devices static profile and dynamic profile using RESTful URIs, and it maintains the N-screen repository and device active status for N-screen aware recommendations. Unlike the conventional recommender systems which can recommend the content the user watched at night time to similar users during office hours, we introduce temporal information to find out which user likes which content at which time to improve the precision, recall, and scalability. The hybrid framework incorporates and fuses various data about the user to enhance the sparsity and cold start issues; that is, the proposed system considers users similarities of content multirating, N-screen attributes, and demographics. Finally, good results in terms of accuracy and precision/recall are achieved by using individual feature and subspace weight based clustering and temporal information. The proposed recommender system can be applied in any field of recommendation, but it is highly applicable in the field of IPTV programs personalization or online movies recommendation because it considers the user N-screen devices profile with its preferences and demographics.

## Figures and Tables

**Algorithm 1:**
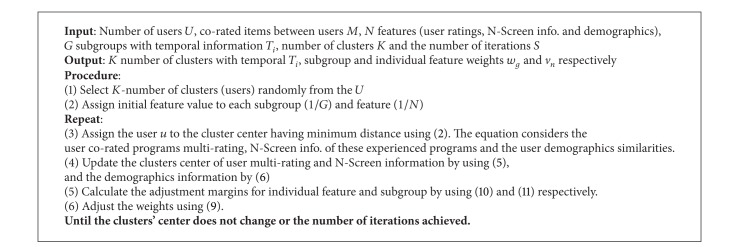
Individual feature and subspace weight based clustering (IFSWC) algorithm.

**Algorithm 2:**
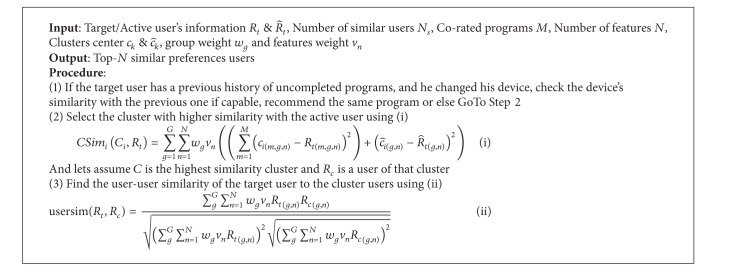
Finding similar preference users to the target/active user.

## Nomenclature

*U*: Number of users
*K*: Number of clusters
*M*: Users corated items/programs
*N*: Number of features (multirating, N-screen information, and demographics)
*λ*_*u*,*k*_: Cluster membership function
*G*: Number of subspaces, that is, subgroups
*v*_*n*_: Weight of a feature *n*
*w*_*g*_: Weight of a subspace *g*
*C*: Centroid, that is, cluster center
*x*: User information vector (multirating and N-screen information)
$\hat{x}$: User demographics
*c*: Cluster user information vector (multirating and N-screen information)
$\hat{c}$: Cluster user demographics
*a*_*n*_: Separation (distance) within cluster objects
*b*_*n*_: Separation (distance) between clusters centroids
*z*: Global mean value
||*c*_*k*_||: Number of users in cluster *k*
*S*: Iteration for cluster objective function converage
*R*_*t*_: Target/active user rating and N-screen information
${\hat{R}}_{t}$: Target/active user demographics
$\bar{R_{t}}$: Average rating of target user.
