# Erratum to “N-Screen Aware Multicriteria Hybrid Recommender System Using Weight Based Subspace Clustering”

**DOI:** 10.1155/2015/261862

**Published:** 2015-06-17

**Authors:** Farman Ullah, Ghulam Sarwar, Sungchang Lee

**Affiliations:** Department of Information & Communication, Korea Aerospace University, Goyang 412-791, Republic of Korea

In the published paper titled ‘‘N-Screen Aware Multicriteria Hybrid Recommender System Using Weight Based Subspace Clustering,” in Figure  2, it was not “Device pynamic profile,” it is “Device dynamic profile” and it was not “inforomation” it is “information.” Also, in Algorithm 2, there was an issue with square root placement in Equation (ii). The right equation is as follows:
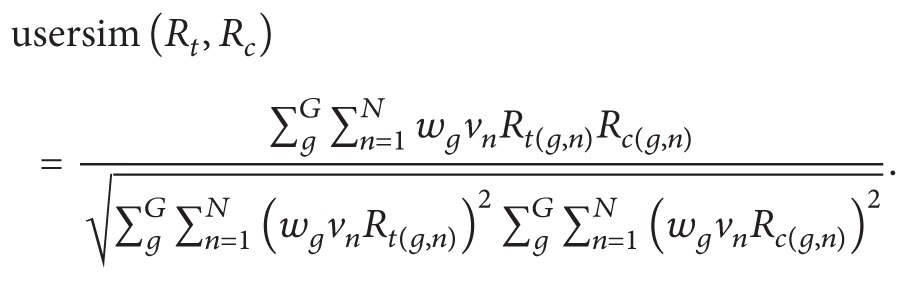
(ii)


## Figures and Tables

**Figure 2:**
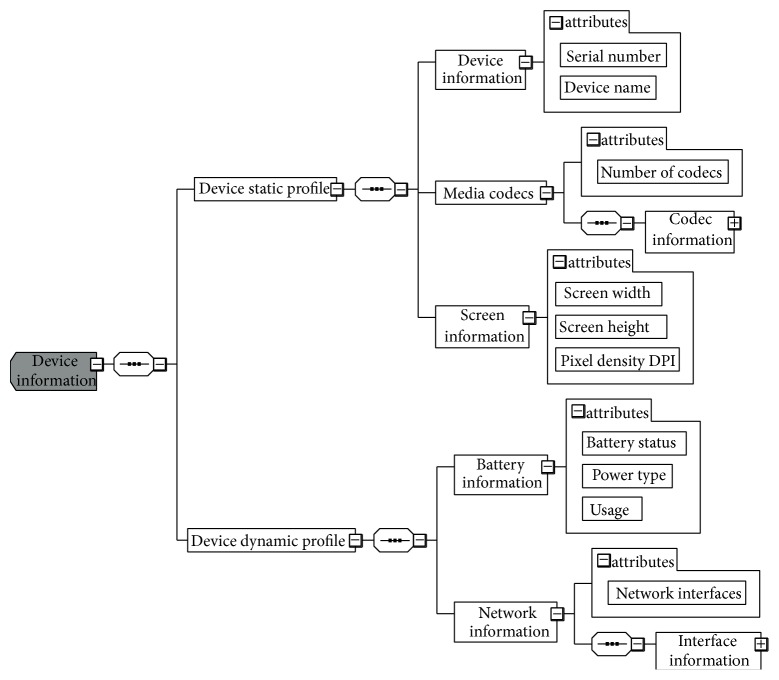
Schema for user N-screen device profile attributes.

